# *Murdannia loriformis*: A Review of Ethnomedicinal Uses, Phytochemistry, Pharmacology, Contemporary Application, and Toxicology

**DOI:** 10.1155/2021/9976202

**Published:** 2021-07-05

**Authors:** Intan Soraya Che Sulaiman, Azham Mohamad, Osumanu Haruna Ahmed

**Affiliations:** ^1^Faculty of Applied Sciences, Universiti Teknologi Mara, Perlis Branch, Arau Campus, Shah Alam 02600, Perlis, Malaysia; ^2^Centre of Foundation Studies for Agricultural Science, Universiti Putra Malaysia, Serdang 43400, Selangor, Malaysia; ^3^Department of Crop Science, Faculty of Agriculture and Food Sciences, Universiti Putra Malaysia Bintulu Campus, Bintulu 97008, Sarawak, Malaysia; ^4^Institut Ekosains Borneo, Universiti Putra Malaysia Bintulu Campus, Bintulu 97008, Sarawak, Malaysia

## Abstract

This review provides an updated and comprehensive overview on the ethnomedicinal use, phytochemistry, pharmacology, and toxicology of *M. loriformis*. Phytochemical analysis of *M. loriformis* revealed that it is composed of phenolics, flavonoids, condensed tannins, chlorophylls, alkaloids, and steroids. Numerous compounds including syringic acid, *ß*-*O*-D-glucopyranosyl-2-(2′-hydroxy-Z-6′-enecosamide) sphingosine, isovitexin, and 3*β*-*O*-D-glucopyranosyl-24*ξ*-ethylcholest-5-ene have been identified and isolated from this plant species. The present review attempts to bridge the gap between traditional use and pharmacological studies of *M. loriformis* while improving their existing therapeutic agents and product applications based on this plant.

## 1. Introduction

Safety is one of the major concerns in the drug development. According to the Food and Drug Administration (FDA), to bring a new pharmaceutical drug to the market, the lead compounds in drug discovery will have to get through drug evaluation process that includes preclinical and clinical phases [1]. Plant kingdom serves as abundance and safe source for new drug discovery. The analysis reported from 1981 to 2019 by Newman and Cragg (2020) revealed that approximately 23.5% sources of new and approved drugs by FDA for the treatment of human diseases are from natural sources such as 11.5% from natural product mimics and 3.2% from total synthesis, but the pharmacophore is still from a natural product [2]. Thus, we suggest that the natural product continues to play a major role in the drug discovery and development process.

Plants survive in an environmental stressor with limitation on temperature, light, water, and nutrient availability by biosynthesizing their specific secondary metabolites. These metabolites enable plant growth, reproduction, and defense against environmental threats [3, 4]. In this manner, a plant may possess various biological activities because of the secondary metabolites in individual plants. This could lead to their potential used as therapeutic agents with anticancer, antioxidant, anti-inflammatory, analgesic, antiaging, antiviral, antipyretic, and antimicrobial properties. *Murdannia loriformis* (Hassk.) Rolla Rao et Kammathy (*M. loriformis*) is among the popular folk medicines consumed by cancer and diabetes patients in Thailand [5, 6]. This plant has gained much attention in Thailand after intake of *M. loriformis* juice. It is reputed for retarding cancer progression besides enabling symptomatic relief resulting from modern therapy [7, 8]. *M. loriformis* is also one of the traditional Chinese remedies used to treat detoxification and respiratory tract complaints [8]. Owing to their therapeutic potential, several scientific reports have been documented to support the claims [7, 9–16]. This review provides a comprehensive summary of the updated information of ethnomedicinal use, phytochemistry, pharmacology, and toxicology of *M. loriformis*. Therefore, the present review may bridge the gap between traditional use and pharmacological studies of *M. loriformis* in addition to improving the existing therapeutic agents and product application of *M. loriformis.*

The literature was searched from different databases using electronic search engines: Google Scholar, Science Direct, PubMed, CAB Direct, Springerlink, MEDLINE Complete, SAGE Journals Online, Ovid LWW, Scopus, Wiley Online Library, EBSCOhost, JetP, thesis, recognized books, and conference proceedings up to March 2021. The keywords used were “*Murdannia loriformis*,” “Beijing grass,” and “Angel grass.” The search was limited to articles published in Malay and English languages. The scientific name of *Murdannia loriformis* was validated using International Plant Name Index (http://www.ipni.org) and The Plant List (http://www.theplantlist.org) databases.

## 2. Botany

**Figure 1:**
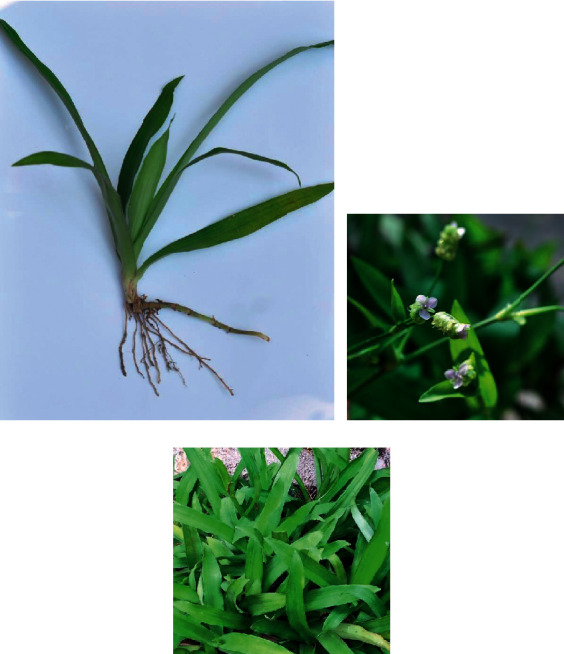
*Murdannia loriformis* (Hassk.): (a) whole plant including roots; (b) flowers; (c) leaves.

*Murdannia loriformis* (Hassk.) Rolla Rao et Kammathy (*M. loriformis*) belongs to the family Commelinaceae, and it originated from India [16]. The plant species is distributed in the forests and grassy slopes of Asia, particularly China and Thailand [17]. This plant is attracting attention in Malaysia for its medicinal potential such as psoriasis and eczema [18]. *M. loriformis* is conspecific to *M. nudiflora* because of their similarity in habit, inflorescence, and capsule. However, *M. loriformis* differs from *M. nudiflora* as follows: sheaths of cauline leaves ciliate only on 1 side of mouth, main stem undeveloped, seeds finely white, reticulate, neither pitted nor verrucose, and pedicels slightly curved. *M. loriformis* is known as “Beijing grass,” or “Angel grass” in English, “Rumput Cina,” “Rumput Beijing,” and Rumput Siti Khatijah in Malay and “Ya Pak King” in Thai (Figure 1) [12, 18, 19].

It is suggested in The Plant List [20] and Flora of China [17] that plants of *Aneilema angustifolium*, *Aneilema loriforme*, *Aneilema terminale, Murdannia angustifolia*, and *Aneilema nudiflorum* are synonymous with *M. loriformis*. Previously, the genus of *Murdannia* Royle was categorized under the genus of *Aneilema*, a large and heterogeneous group. However, after revision of *Aneilema* genus, *Murdannia* has been split off from the genus because of their differences in capsule, flower, and staminode structure [21].

Taxonomical of *M. loriformis* can be classified as follows:


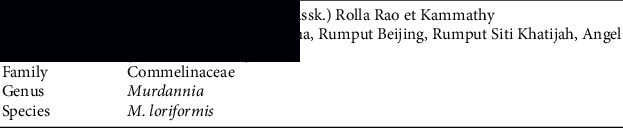


*M. loriformis* is a monocotyledonous and perennial herb. It is approximately 10 cm tall. The basal leaves of *M. loriformis* are simple, blades linear, alternate, and glabrous. The main stem is undeveloped; fertile stems are numerous, arising from the rosette, diffuse or ascending, measure 15–50 cm, glabrous or puberulent on one side, extremely rarely densely hirsute. The basal leaves with a linear blades measure 5–15 cm long and 6–9 mm wide. The flowers are terminal, inflorescence and densely panicle. The involucral bracts are leaflike but smaller than leaves, distal ones extremely small, less than 10 mm, and peduncle about 2.5 cm. Pedicels slightly curved, translucent bracts about 4 mm, sepal, ovate-elliptic, about 3 mm, herbaceous. The petals are blue or bluish violet and obovate orbicular. The capsule is ovoid globose and trigonous, about 3–4 mm. The seeds have two per valve: yellow-brown, radiate, striate, and finely white reticulate. The rhizomes are absent. The roots are fibrous that measures approximately 0.5–1 mm in diameter, glabrous or tomentose. The flower season is between May and October [8, 17].

## 3. Ethnomedicinal Uses

The plant *M. loriformis* is used traditionally for self-treatment by cancer patients among rural communities in Thailand. Different cancer patients have claimed that consuming fresh drinks of *M. loriformis* could prolong life and reduce the adverse effects of radiation and chemotherapy [8, 11]. In Thai folklore medicine, *M. loriformis* has extensively been used as pain relief from bronchitis, diabetes treating, laxative, and cooling agent [5]. Sometimes, the whole plants are decoction with water and taken orally for hepatomegaly treatment [22]. The leaf parts of *M. loriformis* are often used to treat various ailments (Table 1). Their wide use has led to the formulation of many commercial products in the market based on *M. loriformis*, including cream [31], gel [32], capsule [33, 34], tablet [35], tea [36], gummy [37], and mask [38] for cosmetic, nutraceutical, and pharmaceutical purposes.

## 4. Phytochemistry

The phytochemical screening of *M. loriformis* revealed that it is has phenolics, flavonoids, condensed tannin [39], and plant membrane lipid [40] in this plant's extract. With extraction condition of 30 min at 70°, the antioxidant activity and total phenolic content of *M. loriformis* were 30.43 mg TE/mL and 25.52 mg GAE/mL, respectively [41]. Pongsathorn and co-workers quantified chlorophyll *a* (47.88 ± 7.01 *µ*g/g dw) and chlorophyll *b* (50.78 ± 3.89 *µ*g/g dw) in the methanolic extract of *M. loriformis* [42], but carotenoid pigment was not detected. Another phytochemical screening by Phattalung et al. detected phytoconstituents, namely, alkaloids, and steroids in the whole plant extract of 95% ethanol *M. loriformis* [43]. Antioxidant activity of *M. loriformis* measured by DPPH radical scavenging activity was 91.5% at a concentration of 500 *µ*g/mL [42].

**Figure 2:**
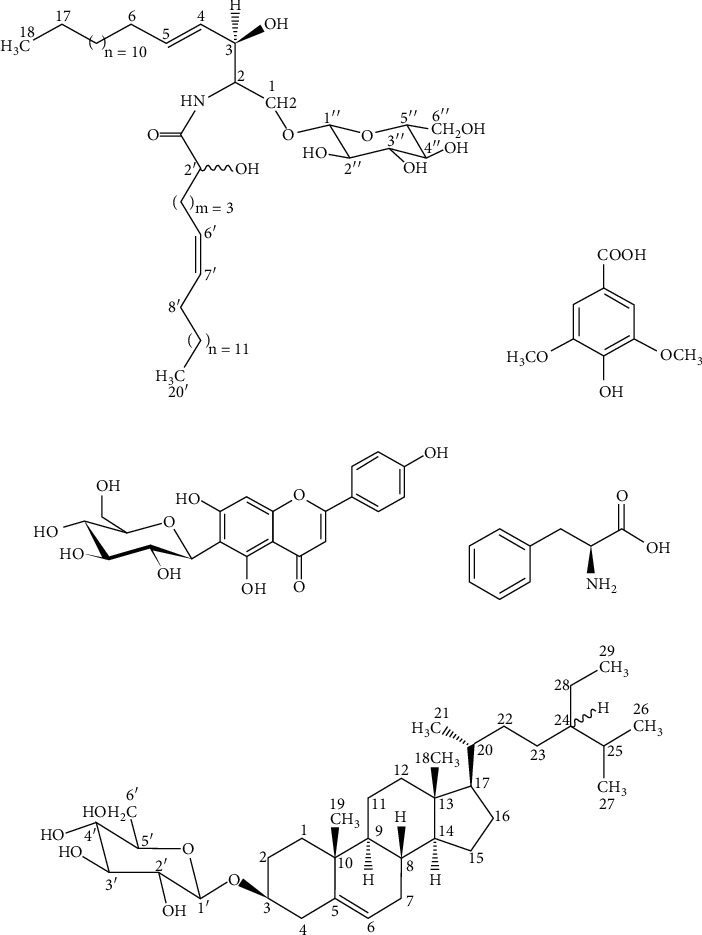
Phytochemicals identified and isolated from *M. loriformis*. (a) *ß*-O-D-Glucopyranosyl-2-(2′-hydroxy-Z-6′-enecosamide). (b) Syringic acid. (c) Isovitexin. (d) Phenylalanine. (e) 3*β*-O-D-Glucopyranosyl-24*ξ*-ethylcholest-5-ene.

Jiratchariyakul et al. successfully isolated phytosterol glucosides, glycosphingolipid (Figure 2(a)), and digalactosyl diglyceride from methanol fraction of ethanolic extract *M. loriformis* plant [7]. This fraction and their pure compound of glycosphingolipid, namely, *ß*-*O*-D-glucopyranosyl-2-(2′-hydroxy-Z-6′-enecosamide) sphingosine have moderate cytotoxic activity against breast and colon cancer cell lines. Further isolation of water-methanol fraction from the same extract established the presence of chalconoid substances, syringic acid (Figure 2(b)), and isovitexin (Figure 2(c)). According to the study, the aqueous fractions of the ethanolic extract of *M. loriformis* have inorganic salt (Na, K, and Mg) and phenylalanine (Figure 2(d)) [7]. Phenylalanine is a type of aromatic amino acids in the plant for protein synthesis besides serving as precursors for the biosynthesis of flavonoids through shikimate pathway resulting in the formation of phenolic compounds such as *trans*-Cinnamic acid, *p*-coumaric acid, condensed tannins, and anthocyanins. [44]. Also, a steroidal glucoside, namely, 3*β*-*O*-D-glucopyranosyl-24*ξ*-ethylcholest-5-ene (Figure 2(e)) has been found in this plant, although these compounds may have no activity to cancer cell lines [11].

Pinitsoontorn et al. [26] quantified the contents of oxalate (89.50 ppm/g dry wt.) and calcium (54.67 ppm/g dry wt.) in *M. loriformis* leaves. However, because this plant is consumed freshly or as herbal tea, further research should focus on their toxicity level of oxalate content related to their safety concern because high levels of oxalate will risk to calcium oxalate kidney stone problem.

## 5. Pharmacological Effects

Several scientific reports support the traditional use of *M. loriformis* in treating different ailments [7, 10, 12, 13, 16, 26, 42, 45, 46]. Although some may not directly correlate with their traditional uses, they provide insight into its therapeutic potential and bioactive properties. The antioxidant, anticancer, antimutagenicity, anti-inflammatory, antimicrobial, and immunomodulatory effects of *M. loriformis* extract are summarized in Table 2.

### 5.1. Antioxidant Activity

The earliest study on the antioxidant activity of this plant was reported by Pinitsoontorn and co-workers [26]. Antioxidant screening studies of over 20 different Thai herbal teas including *M. loriformis* revealed that the antioxidant activity of the tea and its phenolic content was among the average compared to other commercial teas. Pongsathorn [42] further studied the content of phenolics and pigments from methanol extract of *M. loriformis* to understand their antioxidant activity. The results revealed that the phenolic content of *M. loriformis* extract was less <10 mg GAE/g extract, and their chlorophyll *a* and chlorophyll *b* contents were 47.88 and 50.78 *µ*g/g dry weight, respectively. The extract had non-carotenoids. Adisakwattana et al. reported phenolic content in aqueous extract of *M. loriformis* as 12.2 mg GAE/g extract [51].

Techaratanakrai et al. further studied the antioxidant activity of *M. Loriformis* with a focus on the relation to temperature and diffusion time using DPPH assay method [41]. According to Techaratanakrai et al., the antioxidant activity of *M. Loriformis* demonstrated a contrasting effect on total phenolic content regarding infusion time and temperature. This study revealed that the phenolic groups have no significant effect on the antiradical activity measured by the DPPH radical scavenging activity assay; rather, the influence was contributed by other groups of antioxidant contributors such as chlorophyll. According to the authors, the optimum parameter suggested for the extraction condition was 70°C for 30-minute infusion time to obtain the highest antioxidant activity of 30.43 mg TE/mL and a total phenolic content of 25.52 mg GAE/mL [41]. Kittipongpittaya et al. [12] studied the potential benefit from antioxidant biological activity of *M. loriformis* extract in food systems. The results demonstrated that 200–800 ppm of *M. loriformis* extracts applied to pork lard and soybean oil reduced the rate of peroxide values in the oil dependently over 14 days of storage time.

### 5.2. Antimutagenicity and Anticancer Activity

The chemopreventive effects of *M. loriformis* were initially demonstrated by Rearungchom in 1993 [46] with a short-term antimutagenic effect of methanolic extract of *M. loriformis* on aflatoxin B1 (AFB_1_) mutagenesis. During preincubation technique using *Salmonella typhimurium* TA 98 and TA 100 with and without metabolic activation, the tested extract did not demonstrate any mutagenic activity towards AFB_1_ but demonstrated inhibitory effect towards the respective carcinogen (AFB_1_).

Further *in vivo* studies were conducted by Vinitketkumnuen et al. to determine the levels of serum aflatoxin–albumin (AF–albumin) adduct formation in rats after exposure to AFB_1_ [16]. AFB_1_ is a potent fungal toxin that initiates carcinogenesis in human and animal liver [52]. In this chemopreventive study, the rats treated with 3 g/kg body weight (bw) of *M. loriformis* extract were treated with single and multiple doses of 250 *µ*g/kg bw AFB_1_. The results suggest the ability of *M. loriformis* extract to influence the formation of AF–albumin adduct, whereas the rats treated with a single dose of *M. loriformis* extract appeared to modulate the rate of AF–albumin adduct formation. However, multiple doses of the treatment demonstrated slight decrease in the AF–albumin adduct levels. This was probably due to long-term intervention with *M. loriformis* extract through AFB_1_ exposure. This might have caused formation of enzymes associated with carcinogen detoxification, leading to diminishing rates of AFB_1_ formation [53].

An extended chemopreventive study by Intiyot et al. described the antimutagenic effects of the extract towards mutagens and their inhibitory effects on azoxymethane-induced DNA methylation and aberrant crypt focus (ACF) formation in male rats [10]. From the results, *M. loriformis* extract demonstrated antimutagenic activity against the tested heterocyclic amines in dose-dependent manner and decreased the number of ACF (with more than 3 aberrant crypts per focus) formation. ACF with more than three crypts per focus was reported to be associated with precolorectal cancer development [10, 54]. Thus, treatment with *M. loriformis* extract could prevent azoxymethane-induced aberrant crypt foci by acting as a suppressing or blocking agent in the progression of carcinogenesis.

*M. loriformis* extract had been reported to induce DT-diaphorase (DTD) activity when tested on a murine hepatoma cell line (Hepa 1c1c7) [55]. DTD is an anticarcinogenic enzyme that acts as bioreductive agent for protecting tissues against carcinogens, mutagens, and cytotoxics. DTD in animals and humans tissues prevents free radicals formation [56]. In agreement with anticancer, studies by Koontongkaew and co-workers (2009) demonstrated the antiproliferative effectiveness of *M. loriformis* extract against colon and human breast cancer cell lines. Isolated compound of *M. loriformis* extract, namely, glycosphingolipid *β*-*O*-D-glucopyranosyl-2-(2′-hydroxy-Z-6′-enecosamide) sphingosine, has cytotoxic properties at concentrations of 8, 14.5, 12, and 25 *µ*g/mL against human breast, colon, lung, and liver cancer cell lines [7, 11]. Hence, cytotoxic effects of *M. loriformis* discussed earlier scientifically supported the used of the extract in herbal tea consumption for cancer treatment and detoxification purposes practiced by folk medicine.

Moreover, the phenolic compounds such as isovitexin and syringic acid in the extract of *M. loriformis* may also attribute to their chemopreventive and anticancer effects [57, 58]. According to Leon-Gonzalez et al. [58], oxidative stress increases proteins, lipids, and DNA damage in mice to induced colon carcinogenesis. Oxidative stress results in excessive levels of free radicals in humans. Plants with phenolic compounds or polyphenols have been recognized to have high levels of antioxidant activity and marked effects in the prevention of oxidative stresses [59, 60]. Interestingly, in addition to their beneficial effects on antioxidant properties, there are significant evidences to suggest that prooxidant features of polyphenols contribute to their tumoricidal effects [61]. This is related to their ability to alter cellular redox status, whereas, in healthy cells, they act as antioxidant agents to prevent carcinogenesis, but, in cancer cells, they serve as prooxidants to destroy cancer cells [58].

### 5.3. Anti-Inflammatory Activity

Cumulative evidence suggests that inflammation may play a significant role in the development of precancerous lesions; hence, chronic inflammation is reported to increase risk for many cancers [62]. In other words, plant anticancer property may also exhibit anti-inflammatory activity, because of the associated factors of chronic inflammation such as disruption of DNA repair pathways, immunosuppression, and tissue destruction contribute to progression of carcinogenesis [63].

*M. loriformis* has been traditionally used to treat wound [11]. The scientific investigation of *M. loriformis* anti-inflammatory activity has been extensively studied by Kunnaja and co-workers [13]. The authors used carrageenan and arachidonic acid-induced hind paw edema models and cotton pellet-induced granuloma formation and transudation in rats for the acute and chronic inflammatory model, respectively. The finding demonstrates antiedematous effect of the extract in the tested assay through the inhibition effect of the extract towards paw edema in a concentration-dependent manner. M. *loriformis* extract responds to inflammation of rat paw in biphasic events. In the first few hours after injection, the extract suppressed histamine, kinins, and serotonin release. At the second phase, approximately 2.5–6 h later, acute inflammation occurred through the release of prostaglandins by cyclooxygenase (COX) pathway [13, 64]. It is hypothesized that treatment with *M. loriformis* extract may inhibit prostaglandin synthesis and block the cyclooxygenase pathway, whereas arachidonic acid can prevent the release of the two types of proinflammatory mediators, namely, prostaglandins and leukotrienes through dual inhibitors of COX and lipoxygenase (LOX) [65]. A comparative study of *M. loriformis* crude ethanolic extract and its fractions (hexane, chloroform, and ethyl acetate) have demonstrated that hexane fractions possesses stronger effects on nitric oxide reduction such as standard (L-NAME and indomethacin), and it is capable of reducing COX-2 protein expression in LPS-stimulated RAW264.7 cells [66]. Thus, we suggest that the active ingredient of anti-inflammatory of *M. loriformis* is the nonpolar compound [66].

Furthermore, studies on chronic inflammation assay using cotton pellet-induced granuloma formation and transudation in rats suggest that the extract of *M. loriformis* at a concentration of 400 mg/kg has the ability to control the alkaline phosphatase level in serum to a normal level. The inflammatory response of treated rats in the assay involving three phases, transudative, exudative, and proliferative phase resulted in decreased granuloma formation and transudation. Granuloma is a typical feature occurring in the early stage of chronic inflammatory process [67]. Thus, the inhibition effects of *M. loriformis* extract towards granuloma formation and transudation in rats suggest their ability to prevent the proliferative phase of chronic inflammation. Moreover, the extract demonstrated no significant effect on the gastric mucosa of rats when compared to control, indicating that the plant extract possesses desirable anti-inflammatory properties without ulcerogenic effect [14].

### 5.4. Immunomodulatory Effect

In principle, inflammation is a part of the immune response by preventing infection through production of proinflammatory cytokines and formation of inflammatory mediators, while anti-inflammatory activity of *M. loriformis* extract may attribute to its immunomodulatory properties because of the potential activation of immune effector in peripheral blood mononuclear cells (PBMC) [11]. In supporting one of its folklore uses for strengthening immune system [29], the literature is replete with evidence that *M. loriformis* extract improves immune system. Herb juice of *M. loriformis* extract using water contains glycosphingolipid *β*-*O*-D-glucopyranosyl-2-(2′-hydroxy-Z-6′-enecosamide) sphingosine. These compounds are reputed for increasing PBMC proliferation. This suggests the potential mitogenic properties of the extract [11]. In a comparative study, the researchers also reported that pure compound of glycosphingolipid was more effective than herb juice extract in stimulating PBMC proliferation. Both increase the expression of CD 3,4 molecules in T lymphocytes. However, in contrast to what has been reported by Punturee et al. [24], the ethanolic and water extract of *M. loriformis* at higher concentrations decreased T- and B-cell proliferation, which may be toxic to human PBMCs. Thus, the toxicity effects of *M. loriformi*s extract on nonspecific cellular immune responses warrant further investigation.

### 5.5. Antipyretic Activity

Scientific reports justify the ethnomedicinal use of *M. loriformis* for fever treatment because of its antipyretic effect [13]. In this study, rats receiving *M. loriformis* extract at 400 mg/kg had their rectal temperature reduced to normal in 30 min and the effect persisted for 180 min.

### 5.6. Analgesic Activity

In another study, Kunnaja et al. reported the potential analgesic effect of *M. loriformis* extract in dose-dependent manner to reduce paw licking time of the rats at both phases of formalin test [13]. The test involved two distinct phases, early (neurogenic) and late (inflammatory) phases, and it was carried out to check the effects of the extract on inflammatory pain. Analgesic action of the extract to reduce paw licking time in both phases suggested the extract property as an opioid drugs to produce morphine-like effect for pain relief [68]. Moreover, several researchers had reported the contribution of glycosides, alkaloids, tannins, and flavonoids contained in plants for analgesic action [68].

### 5.7. Antimicrobial and Antibacterial Activity

The antimicrobial activity of *M. loriformis* extract was evaluated against Gram-positive bacteria *Propionibacterium acne* (*P. acnes*) and *Staphylococcus epidermidis* (*S. epidermidis*) [9]. In the study, no bactericidal effect was observed against *P. acnes*; however, the tested extract inhibited the growth of *S. epidermidis* at a minimal inhibitory concentration (MIC) of 1.25 mg/mL [9]. In contrast, Kaewkod et al. [69] reported no antibacterial activity of ethanolic and aqueous extract of *M. loriformis* against *S. epidermidis*. The tested extracts of *M. loriformis* had no antibacterial activity against the other five bacteria causing skin diseases, namely, *S. aureus*, *Bacillus* sp., *Ps. Aeruginosa*, *P. acnes,* and MRSA. However, the ethanolic extract of *M. loriformis* exhibited antibacterial activity against *M. luteus* within the diameter of inhibition zone 7 mm. MIC and minimal bactericidal concentrations (MBC) of ethanolic extract of *M. loriformis* against *M. luteus* were 62.5 and 125 mg/mL, respectively. Meanwhile, aqueous extract of *M. loriformis* inhibited the growth of *Corynebacterium* sp. with the diameter of inhibition zone 20.0 mm. MIC and MBC values of aqueous extract against bacterial strain of *Corynebacterium* sp. were 62.5 and 250 mg/mL, respectively [69]. Both results indicate mild bactericidal activity of *M. loriformis.*

The potential using *M. loriformis* has been tested for light-activated antimicrobial activity against several bacterial and fungi. The ethanolic extract of *M. loriformis* under UV light produced a good inhibition zone (8 mm to 12 mm) against *Bacillus subtilis*, whereas no activity was observed under dark condition [23]. Besides, the extract demonstrated no activity in both conditions against *Staphylococcus aureus*, *Escherichia coli*, *Pseudomonas aeruginosa*, *Candida albicans,* and *Aspergillus fumigatus*. Similar findings by Phatthalung et al. [43], Limsuwan et al. [49], Chomnawang et al. [50], and Kaewkod et al. [69] have reported that *M. loriformis* extract was not effective against *Staphylococcus aureus*. This light-mediated activity of *M. loriformis* extract can be used in the future in the development of photochemistry and photochemotherapy approaches of this plant.

### 5.8. Gastroprotective Activity

The folklore use of *M. loriformis* to relieve inflammation [11, 12] is scientifically supported by their antigastric ulcer activity using three different models: ethanol/hydrochloric acid, indomethacin, and restraint water immersion stress [14]. *M. loriformis* extract in a dose-dependent manner significantly inhibited gastric ulcer formation induced by all tested models [14]. Oral administration of *M. loriformis* extract at 400 mg/kg significantly increased the amount of gastric wall mucus and reduced gastric acid secretion in the pylorus ligation model. In the model, pylorus ligature rats treated with *M. loriformis* extract had significantly reduced total acidity and gastric volume besides increasing gastric pH compared to the control group. Gastric ulcers occur because of imbalance between mucosal defensive mechanisms and endogenous or exogenous aggressive factors [70]. From the findings, *M. loriformis* demonstrates gastroprotective action on ulcer models through reduction of aggressive factor (gastric acid) and increasing of defensive mechanism (mucus). Thus, the growth of gastric wall mucus plays an important role as a defensive factor against gastrointestinal damage.

### 5.9. Other Activities

*M. loriformis* extract inhibits pancreatic lipase activity in dose-dependent manner (IC_50_ = 0.11 ± 0.01 mg/mL), albeit being less potent than the standard orlistat sample (IC_50_ = 1.34 ± 0.13 mg/mL) [51]. From the experiment, *M. loriformis* extract at 2 mg/mL inhibited pancreatic cholesterol esterase activity by 10% to 22% (IC_50_ > 3 mg/mL). Nonetheless, the tested extract was less potent compared with control, simvastatin (IC_50_ = 0.08 ± 0.01 mg/mL) [51]. In the *in vitro* cholesterol solubilization assay using artificial micelles, *M. loriformis* extract moderately inhibited cholesterol micellization by 18%. Furthermore, the extract binds glycodeoxycholic acid and taurocholic acid by 30% and 28%, respectively. However, taurodeoxycholic acid binds the extract by 6% [51]. The reason for using *M. loriformis* extract for antihyperlipidemic activity is based on delaying lipid digestion and absorption through gastrointestinal mechanisms. The mechanisms involve inhibition of pancreatic lipase, pancreatic cholesterol esterase activity, inhibition of cholesterol micellization, and bile acid binding [51]. Several studies had suggested that plant polyphenols have lipid lowering potential [71, 72]. Daily intake of polyphenol-enriched edible plants such as *M. loriformis* extract may delay the increase of postprandial hypertriacylglycerolemia and hypercholesterolaemia in obese individuals. Hence, the extract has been identified as a potential hypolipidemic agent for preventing and treating hyperlipidemia [51].

A previous study has validated the traditional use of *M. loriformis* extract for diabetes mellitus because of its antihyperglycemic activity. *M. loriformis* extract is a potent antidiabetic agent, which improves blood glucose level in diabetic patients [12, 39]. At 1 mg/mL, the respective plant extract markedly inhibited 34.63% of glycation in fructose-mediated nonenzymatic glycation at early week [39]. *M. loriformis* extracts demonstrated moderate pancreatic *α*-amylase and intestinal *α*-glucosidase, maltase, and sucrose inhibition with IC_50_ values of 0.86, 3.43, and 3.46 mg/mL, respectively. In the study, *M. loriformis* extracts had 12.2 mg/g of total phenolic content followed by flavonoids 4.34 mg/g and condensed tannins at 100.7 mg/g, which indicates the potential of *M. loriformis* antidiabetic properties. Phenolic, mainly flavonoids, rich extracts have better *α*-glucosidase inhibitory activity that controls blood glucose and type 2 diabetes [73–75].

## 6. Toxicity

Acute toxicity study for 14 days after oral administration of *M. loriformis* extract at 5000 mg/kg body weight did not demonstrate any signs of mortality [13]. Tested rats similarly demonstrated general behavior and physiological activities in comparison to the control group. No abnormalities and pathological changes were observed in terms of size and color of internal rat organs suggesting that the *M. loriformis* extract was not toxic at 5000 mg/kg. However, the water extract of *M. loriformis* decreased T- and B-cell proliferation with or without mitogen [24]. Hence, further studies should be carried out on their toxicity effect on nonspecific cellular immune responses to justify the safety of *M. loriformis* extract in humans.

## 7. Conclusions and Future Perspectives

It is well documented in the literature that the polar extract of the whole plant of *M. loriformis* is the most studied part of this plant. Biological activity of polar extracts is associated with their phenolic compounds. Phenolic compounds, which are synthesized by plants for their own protection against pests, could also be used in humans as an antioxidant. Antioxidant has many biological functions in the human body to enable preventing various diseases. Polar extracts of *M. loriformis* possess a wide range of pharmacological actions, such as antioxidant, antimutagenicity, anticancer, anti-inflammatory, immunomodulatory effects, antipyretic, analgesic, antimicrobial, antibacterial, and gastroprotective activity that support its folk use.

Although several reports might justify the ethnomedicinal use of *M. loriformis*, the existing data are insufficient, and there is a need for further *in vitro* and *in vivo* pharmacological models, as well as clinical studies. Yet, only a few acute toxicology studies report polar extracts of this plant. Future consideration should be on subacute and chronic toxicity tests for other polarity extracts of this plant as well (semipolar and nonpolar). Because *M. loriformis* plants traditionally consume fresh or decoction with water, it is essential to ensure that the intake is kept below tolerance levels. Safer dosage limits for human consumption and effects on the target organs toxicity such as lung, liver, kidney, and heart should be clarified and clearly evaluated.

To date, knowledge on the phytochemical constituents of *M*. *loriformis* is limited. Therefore, further research is needed for phytochemistry analysis of *M*. *loriformis* for better understanding of the mechanisms underlying the bioactive constituents and its bioactivity. Future research should employ sophisticated chromatography and spectroscopic techniques such as supercritical fluid chromatography, LC-NMR, and LC–QTOF over the standard analysis methods.

Moreover, consideration should be given to extraction techniques including extraction time, temperatures, and solvent type and ratio to optimize extraction yields in addition to enhancing the interest components. Hence, a systematic design of optimization process using effective tools such as response surface methodology and mixture design may offer many advantages over conventional optimization method, which is tedious, lacks data evaluation, and is expensive. Future research should put emphasis on biological studies on a particular part of the plant such as leaves, flowers, and roots because the existing data focus on the whole part of *M. loriformis*.

It is hoped that this present review bridges the gap between traditional use and pharmacological studies of *M. loriformis*, hence, providing basic information with significant directions for future researchers in developing therapeutic agents and product applications based on this plant.

## Figures and Tables

**Table 1:** Ethnomedicinal uses of *M. loriformis.*

Indications	Plant part used	Herbal preparation	Prescription and dosage form	Reference
Cough, flu and allergies	Fresh leaves	NAD	NAD	[23]
Hemostatic and diuretic remedies, antioxidant and anticarcinogen	Fresh aerial plant	Fresh juice	Take orally, twice a day (Morning and night)	[7]
Detoxification and respiratory tract complaints	NAD	NAD	NAD	[11]
Cooling effect, laxative	NAD	NAD	NAD	[5]
Pain relief from bronchitis, antimutagenic	Whole fresh plants	Extraction with 80% ethanol	NAD	[16, 24]
Diabetes mellitus, throat infections, pneumonia and inflammation (inflamed wound)	NAD	NAD	NAD	[11, 12, 25]
Cures lymphadenopathy	NAD	NAD	NAD	[26]
Cancer treatment, chemopreventive effects	NAD	NAD	NAD	[16, 27, 28]
Strengthen immune system, fever, ulcer and to treat colon and breast cancer	Fresh leaves	Blend 20–25 of fresh leaves with apple in 1 glass of water. Grind 20–25 of fresh leaves, filtered	Take orally, 1 glass, 2-3 times a day or 2 tablespoon (concentrated) or 30 ml (diluted), 2-3 times a day	[29]
Psoriasis and eczema	Fresh leaves	Blend 6 of fresh leaves with 4 tablespoons of water, filtered	Take orally, twice a day (morning and night)	[30]
Kidney stones relief and kidney detox	Whole fresh plant (include root)	Decoction 7–9 of whole plants with 1.5 mL water	NAD	[30]
To tenderize meet	Fresh leaves	Tied in a knot, placed in cooking	NAD	[30]

NAD: Not appropriately described.

**Table 2:** Pharmacological studies on *M. loriformis.*

Activity tested	Plant part	Extract	Experimental procedures	Dosage concentration	Results	References
*Antioxidant*	Whole plant	80% ethanol	1,1-Diphenyl-2-picrylhydrazyl (DPPH) assay.	200–800 ppm	Showed a concentration-dependent DPPH radical scavenging activity.	[12]
	Leaves	Deionized water	Determination of peroxide values according to AOCS method.	0–800 ppm	Rate of peroxide values in pork lard and soybean oil containing *M. loriformis* have been reduced.	[12]
			Ferric reducing antioxidant power (FRAP) assay.	NAD	Antioxidant activity and total phenolic content of *M. loriformis* extract was 4.14 mM Fe (II)/g dry wt and 46.96 mg GAE/g dry wt, respectively.	[26]
			Determination of total phenolic content using Folin-Ciocalteu method.	NAD	Increased temperature of extraction extremely decreased the DPPH radical scavenging activity.	
	NAD	Water	Investigation the effect of time and temperature on antioxidant activity of *M. loriformis* by DPPH assay.	NAD	Increased time of extraction slightly increased the total phenolic content of the extract.	[41]
			Folin-Ciocalteu method.	NAD	Total phenolic content of the extract was less <10 mg GAE/g extract.	
	NAD	95% methanol	Folin-Ciocalteu method.	0.05–500 *µ*g/mL	Exhibited a concentration-dependent DPPH radical scavenging activity.	[42]
			DPPH assay.	1000 *µ*g/mL	Chlorophyll *a* and chlorophyll *b* were quantified in *M. loriformis* extract.	
			Determination of pigment content according to Lichtenthaler and Wellburn [47].			

*Antimutagenicity*	Whole plant	80% ethanol	*Salmonella* mutation assay	0.1–1.0 g/kg body weight	*M. loriformis* showed antimutagenicity activity by its inhibitory effects on azoxymethane-induced DNA methylation and aberrant crypt focus formation in male F344 rats.	[10, 15]
	Whole plant	Methanol	*Salmonella* mutation assay.	0.05 *µ*g	Methanol extract showed antimutagenicity activity against aflatoxin B_1_ (AFB_1_) mutagenesis in the short term.	[46]
	Whole plant	80% ethanol	Competitive enzyme-linked immuno-sorbent assay (ELISA).	3 g/kg body weight	Multiple doses of treatments have decreased AF–albumin adduct levels.	[16]

*Anticancer*	Whole plant	80% ethanol	MTT assay. Cytotoxicity of *M. loriformis* was determined against breast (MCF7) and colon (HT29) cancer cell lines	20–100 *µ*g/mL	Moderate cytotoxic activity ED_50_ less than 10 *µ*g/mL.	[7]
	Whole plant	95% ethanol	Cytotoxicity of *M. loriformis* was examined against immortalized human keratinocytes (HaCaT), oral epithelial (HN4) and carcinoma cell lines of head and neck squamous (HN12), breast (MCF7) and colon (HT29).	0–10.0 mg/plate	Showed antiproliferative effect on HT29 and MCF7 cells.	[45]
					No effect on HaCaT, HN4 and HN12 cell.	

*Anti-inflammatory*	Aerial part	80% ethanol	Carrageenan- and arachidonic acid (AA)-induced paw edema in rat's assay.	100–400 mg/kg	*M. loriformis* extract significantly reduced the carrageenan-induced edema formation of the rat paw.	[13, 48]
				100–400 mg/kg	Inhibited of AA-induced paw edema in dose-dependent manner.	
			Cotton pellet-induced granuloma formation in rat's assay.	400 mg/kg	*M. loriformis* extract significantly lowered the transudative weight. The inhibitory effect of granuloma formation was well correlated with their transudative weight.	
			Evaluation of the ulcerogenic effect.	400 mg/kg	*M. loriformis* did not affect the gastric mucosa of rats (ulcer index = 0).	

*Immunomodulatory effect*	NAD	Pressed and added of 50–100 mL water. (Herb juice)	*In vitro* cellular immunological assays.	500 *µ*g/mL (herbal juice)	Herb juice and isolated compound namely glycosphingolipid *β*-*O*-D-glucopyranosyl-2-(2′-hydroxy-Z-6′-enecosamide) sphingosine have increased PBMC proliferation in the presence of the mitogen PHA (phytohemagglutinin).	[11]
		Ethanol (isolated compound)		0.01 *µ*g/mL (isolated compound)	Both increase the expression of CD 3,4:CD 3,8 ratio in T lymphocytes.	
	Whole plant	80% ethanol	Lymphocyte activation assay.	1–200 *µ*g/mL	Decrease of T- and B-cell proliferation with the presence and absence of mitogen.	[24]
		Water	Lymphocyte activation assay.	1–200 *µ*g/mL	The water extract significantly decreased PHA and pokeweed mitogen (PWM)-induced lymphocyte proliferation.	

*Antipyretic*	Aerial part	80% ethanol	Yeast-induced hyperthermia assay	400 mg/kg	Reduced the rectal temperature to normal in 30 min and lasted for 180 min.	[13]

*Analgesic*	Aerial part	80% ethanol	Formalin test	20–80 mg/kg	Reduced the licking time in early and late phases.	[12]

*Antimicrobial*	Whole plant	NAD	Disc diffusion and broth dilution methods.	1.25 mg/mL	Have no activity against *Propionibacterium acnes.*	[9]
					Inhibited the growth of *Staphylococcus epidermidis* at MIC value of 1.25 mg/mL. However, the MBC value was >5 mg/mL.	
	Whole plant	95% ethanol	Antibacterial and antifungal assays.	1 mg/mL	Inhibited the growth of *B. subtilis* under the influence of UV light with zone inhibition diameter of 8–12 mm.	[23]
			*M. loriformis* extract was tested for light- mediated activities against *Bacillus subtilis*, *Staphylococcus aureus* K147 methicillin-sensitive (Ms), *Escherichia coli* DC10, *E. coli* (wild), *Pseudomonas aeruginosa* 187 (wild), *Candida albicans* and *Aspergillus fumigatus*.			

*Antibacterial*	Whole plant	Ethanol	Disc diffusion method. *M. loriformis* was investigated against *S. aureus*	0.5–5000 *µ*g/mL	No antibacterial activity.	[43, 49, 50]

*Gastroprotective activity*	Whole plant	80% ethanol	Gastroprotective activity studies using three different models: EtOH/HCl, indomethacin, and restraint water immersion stress.	100–400 mg/kg	*M. loriformis* extract significantly inhibited gastric ulcer formation induced by EtOH/HCl, indomethacin and stress.	[14]
			Gastric visible mucus secretion.	400 mg/kg	Significantly increased the amount of gastric wall mucus.	
			Pylorus ligation.	400 mg/kg	Reduced gastric acid secretion in the pylorus ligation model.	

*Inhibition of pancreatic lipase and pancreatic cholesterol esterase, cholesterol micelle formation and bile acid binding.*	Whole plant	Water	Pancreatic lipase inhibition assay.	NAD	Inhibited pancreatic lipase activity in dose-dependent manner (IC_50_ = 0.11 ± 0.01 mg/mL).	[51]
			Pancreatic cholesterol esterase inhibition assay.	NAD	Inhibited pancreatic cholesterol esterase activity about 10–22% ((IC_50_ => 3 mg/mL).	
			Cholesterol micellization assay.	10 mg/mL	Moderated cholesterol micellization inhibition (18.01 ± 1.44%)	
			Bile acid binding assay.	1 mg/mL	*M. loriformis* extract has bind to glycodeoxycholic acid, taurocholic acid and taurodeoxycholic acid at 30.31 ± 6.88%, 28.70 ± 2.08% and 6.52 ± 0.88%, respectively.	

*Inhibition of α-glucosidase, pancreatic α-amylase and protein glycation activities.*	Whole plant	Water	Phytochemical analysis.	NAD	The total phenolic, flavonoid and condensed tannin content of *M. loriformis* extract were 12.2 ± 2.6, 4.34 ± 0.12 and 100.7 ± 19.0 mg/g extract, respectively.	[39]
			Intestinal *α*-glucosidase inhibitory assay.	NAD	Showed moderate *α*-glucosidase inhibition; IC_50_ maltase (3.43 ± 0.18 mg/mL) and sucrose (3.46 ± 0.04 mg/mL).	
			Pancreatic *α*-amylase inhibition assay.	NAD	The IC_50_ values for *α*-amylase of *M. loriformis* extract was 0.86 ± 0.10 mg/mL.	
			Protein glycation inhibitory assay.	1 mg/mL	Inhibition of glycation in fructose-mediated nonenzymatic glycation by 34.63% at week 1. However, the inhibition percentage slightly decreased at weeks 2–4 (33.02–28.76%).	

## Data Availability

The data used to support the findings of this study are included within the article.
